# Blood Pressure Goals in Critically Ill Patients

**DOI:** 10.14797/mdcvj.1260

**Published:** 2023-08-01

**Authors:** Karuna Puttur Rajkumar, Megan Henley Hicks, Bryan Marchant, Ashish K. Khanna

**Affiliations:** 1 Wake Forest University School of Medicine, Atrium Health Wake Forest Baptist Medical Center, Winston-Salem, North Carolina, US; 2 Outcomes Research Consortium, Cleveland, Ohio, US

**Keywords:** mean arterial pressure, perfusion pressure, hypoperfusion, shock, cardiac function, acute kidney injury, intra-operative hypotension, cardiopulmonary bypass

## Abstract

Blood pressure goals in the intensive care unit (ICU) have been extensively investigated in large datasets and have been associated with various harm thresholds at or greater than a mean pressure of 65 mm Hg. While it is difficult to perform interventional randomized trials of blood pressure in the ICU, important evidence does not support defense of a higher pressure, except in retrospective database analyses. Perfusion pressure may be a more important target than mean pressure, even more so in the vulnerable patient population. In the cardiac ICU, blood pressure targets are tailored to specific cardiac pathophysiology and patient characteristics. Generally, the goal is to maintain adequate blood pressure within a certain range to support cardiac function and to ensure end organ perfusion. Individualized targets demand the use of both invasive and noninvasive monitoring modalities and frequent titration of medications and/or mechanical circulatory support where necessary. In this review, we aim to identify appropriate blood pressure targets in the ICU, recognizing special patient populations and outlining the risk factors and predictors of end organ failure.

## Introduction

Variations in blood pressure above or below the physiological baseline is a common occurrence in the critical care unit, particularly in patients with underlying cardiovascular pathology. The distinction between hypotension and hypoperfusion is particularly critical in the cardiac intensive care unit (CICU), specifically for patients in the perioperative period. The perfusion of an organ is dependent on the mean arterial pressure, which is a function of cardiac output and systemic vascular resistance. A shift in thought process around the definition of shock may be the presence of hypoperfusion, even in the setting of normotension, which is generally thought of as “pre-shock” or compensated shock. Unsurprisingly, outcomes, including mortality, are worse in patients with evidence of both hypotension and hypoperfusion.^1^ In this review, we aim to identify appropriate blood pressure targets in the ICU, recognizing special patient populations and outlining the risk factors and predictors of end organ failure.

## Appropriate Blood Pressure Target in a Critically Ill Patient

Historically, mean arterial pressure (MAP) has defined blood pressure target thresholds in the critical care unit. Every unit of time-weighted average (TWA) spent at MAP < 65 mm Hg has been strongly associated with myocardial infarction, acute kidney injury, and mortality in the critical care unit.^2^ This goal MAP is recommended as the lowest pressure to target initial resuscitation in patients with septic shock by the guidelines of the Surviving Sepsis Campaign.^3^ The extrapolation of the same threshold to all patient types, pathologies, and comorbidities is a challenge and certainly deserves more investigation.

Randomized trials of blood pressure targets are inherently difficult. Asfar and colleagues performed the largest such trial in septic shock patients and compared mean pressures of 80 mm Hg to 85 mm Hg (high target group) with current recommendations of 65 to 70 mm Hg (low target group) in patients with septic shock undergoing resuscitation. The primary outcome was mortality at 28 and 90 days, and they found no difference.^4^ Importantly, pressures were about 5 mm Hg higher in each group, and this was likely a trial of two higher pressures, albeit with adequate intergroup separation.

A recent retrospective analysis from a well-validated electronic ICU dataset with nearly 80,000 patients found that components of blood pressure, namely mean, systolic, and diastolic pressures, were comparable in their strength of association with organ system injury and ICU mortality.^5^ Estimated change-points for the risk of ICU mortality in septic patients were 69 mm Hg for mean, 100 mm Hg for systolic, 60 mm Hg for diastolic, and 57 mm Hg for pulse pressure.^5^ Other observational data derived from large datasets show that higher thresholds of MAP closer to 80 to 85 mm Hg may be the inflection point for increasing harm in both the medical and surgical ICU population.^2,6,7^ While it is difficult to separate intraoperative hypotension from postoperative hypotension in the ICU, even previously normotensive operating room patients suffer harm with new-onset hypotension in the critical care unit.^8^ Thresholds at 75 mm Hg or higher may be associated with decreased delirium and improved arousal levels as measured by sedation scales.^7,9^ In the SEPSISPAM (Sepsis and Mean Arterial Pressure) trial, patients with chronic hypertension had significantly more renal injury if they were exposed to a MAP of 70 to 75 mm Hg (ie, in the lower target group) while more rhythm abnormalities occurred in the higher target group at a MAP of 85 to 90 mm Hg.^4^ Lamontagne and colleagues in the 65-trial showed no difference in survival when patients were randomized to a MAP of at least 65 mm Hg versus permissive hypotension at a MAP of 60 mm Hg.^10^

In the critically ill cardiac patient population, diastolic blood pressure (DBP) is an important determinant of coronary blood flow, and low DBP, particularly < 60 mm Hg, has been associated with increased risk of myocardial injury.^11^ Critically ill patients with cardiogenic shock and a 24-hour average MAP < 65 mm Hg have a rapidly increasing rate of mortality, exceeding 70% with a 24-hour average MAP < 60 mm Hg.^12^ Further, patients with sustained (duration > 10 minutes), MAP < 64 mm Hg before, during, and after cardiopulmonary bypass (CPB), particularly those with sustained MAP < 55 mm Hg, have a strong association with acute perioperative stroke.^13^ Other investigators have confirmed that duration of hypotension per 10 minutes of a MAP < 65 mm Hg during an entire cardiac surgery and after CBP is equally important.^14^

The conundrum, therefore, is whether we should target higher pressures in all patients or in more vulnerable ones. Furthermore, it seems that the mechanisms to achieve higher pressures may be as important as the goal itself. Exposure to supratherapeutic doses of catecholamines may not be desirable.^15,16^ Almost 10 years after the impressive work by the SEPSISPAM group, we need to think of trials with appropriate patient populations, targets, and outcomes. Certainly, the concept of perfusion pressure being the difference of MAP and central venous pressure or in some cases intra-abdominal pressure is a critically important area of focus.^17^ Perfusion pressure may be extrapolated to renal perfusion and a necessary threshold of at minimum 60 mm Hg. Analysis comparing perfusion pressure deficits and MAP deficits has been seen to have a nearly similar and strong association with ICU mortality at two weeks.^18,19^

### Cardiac Critical Care: Different Patient Populations and Personalized Blood Pressure Targets

Individualization of MAP goals is important in the post-cardiac surgery patient, as different comorbidities, operative interventions, and the presence of mechanical circulatory support, among other criteria, may influence the MAP required to maintain adequate perfusion (Table 1).

**Table 1:** Individualized blood pressure management in the post-cardiac surgery patient. SAM: septal anterior motion; CPB: cardiopulmonary bypass; MAP: mean arterial pressure; HCOM/SAM: hypertrophic obstructive cardiomyopathy/systolic anterior motion; LVOT: left ventricular outflow tract; LVH: left ventricular hypertrophy; SBP: systolic blood pressure; VSD: ventricular septal defect; LVAD: left ventricular assist device; VV vs VA ECMO: venovenous vs venoarterial extracorporeal membrane


A. Decision points for individualized blood pressure targets for post cardiac surgery patients

1. Age

2. Nature of surgery

3. Intraoperative course and intraoperative data

4. Left ventricular versus right ventricular dysfunction

5. Presence or absence of mechanical circulatory support devices

B. Patients who need a higher mean arterial pressure goal to optimize tissue perfusion

1. Right ventricular (RV) failure

2. Post-heart transplant with RV dysfunction

3. Hemodynamic instability on weaning from CPB

4. Air in the coronary arteries post-cardiac surgery needing higher MAP goals during initial few hours postoperatively

5. Patients with long CPB time and cross-clamp time

6. Acute kidney injury identified by low urine output intraoperatively

7. HCOM/SAM needing higher MAP to stent open the LVOT

8. Patient with LVH

9. History of chronic hypertension with shift in cerebral autoregulation

C. Patients who need a lower MAP goal to optimize perfusion

1. Patients with LV dysfunction where a reduction in afterload will help increase cardiac output

2. Post aortic surgery, complex congenital repairs, fistula repairs and VSD repairs where a higher-than-normal SBP/MAP can cause increased tension on critical suture sites and lead to catastrophic bleeding

3. Patients with circulatory support devices LVAD, impella, VV vs VA ECMO where increase in systemic arterial blood pressure can compromise flow through these support devices


Most commonly, patients with chronic uncontrolled hypertension are presumed to have a rightward shift in their cerebral autoregulation curve, thereby requiring a higher average MAP goal to maintain cerebral blood flow and perfusion. A frequently referenced threshold for these patients is a minimum MAP no less than 20% from their baseline blood pressure, usually averaged over several previous measurements.^20^ Mechanistically, a worsened microcirculation in the chronically hypertensive and the need for a higher perfusion pressure to maintain end-organ perfusion contribute to the underlying pathophysiology.^21^

After post-acute myocardial infarction, higher MAP goals are associated with smaller infarct sizes, and thus use of vasopressor and inotropic support to obtain higher mean pressures is recommended in this population.^22^

Patients with uncorrected valvular lesions and physiology that requires higher afterload may also require higher MAP goals. These specifically include patients with clinically significant aortic stenosis, left ventricular (LV) hypertrophy, and those with septal anterior motion of the anterior leaflet of the mitral valve due to hypertrophic obstructive cardiomyopathy or asymmetric septal hypertrophy. Finally, patients who develop tamponade physiology postoperatively require higher afterload until the pericardium can be decompressed. Given ventricular interdependence, maintenance of normal to slightly increased systemic blood pressure is critical to maintaining the transseptal pressure gradient and thus normal wall motion of both the left and right ventricles. Consequently, those with significant right ventricular dysfunction, including those post-orthotopic heart transplantation, will require higher MAP goals.^23^

Other specific populations that may benefit from higher MAP goals include patients with coronary air embolism during weaning from CPB,^24^ patients with a prolonged CPB run or cross-clamp time, and those who have difficulty weaning from CPB, which may be associated with relative coronary ischemia. Finally, poor perioperative urine output may indicate end-organ hypoperfusion and increased risk of acute kidney injury, so such patients may benefit from higher perfusion pressure.^25^ In contrast, patients with hemodynamically significant aortic insufficiency and mitral regurgitation require reduced afterload. Lower MAP goals within limits and without compromising on coronary or another end-organ perfusion may be necessary.

Aside from patients with valvular or other pathology, a small subset of patients may have indications for lower MAP goals. Predominantly, this group includes patients who have undergone aortic surgery, those with complex congenital repairs (including fistulas), and those with ventricular septal defects—all cases in which increased MAP leads to tension on critical suture lines, causing possible disruption that can lead to catastrophic and potentially irreparable bleeding. However, recent emerging evidence suggests that this may be an exaggerated association of lower clinical significance.^26^

Another subset of patients in whom decreased systemic blood pressure may be appropriate is those with mechanical circulatory support devices, including temporary and durable LV assist devices, as well as extracorporeal membranous oxygenation through which flow may be compromised in high afterload states.^21^ Appropriate MAP targets in this setting remain a particular enigma since increasing pressure with afterload most surely compromises forward flow in most of these patients.

If relative hypotension is deemed appropriate in a specific patient, close monitoring of end-organ function is mandatory, and indications should be repeatedly addressed to liberalize toward normal MAP goals as soon as it is safe.

### Choosing Pressure versus Indicators of Tissue Perfusion when Managing Shock States

**Figure 1:**
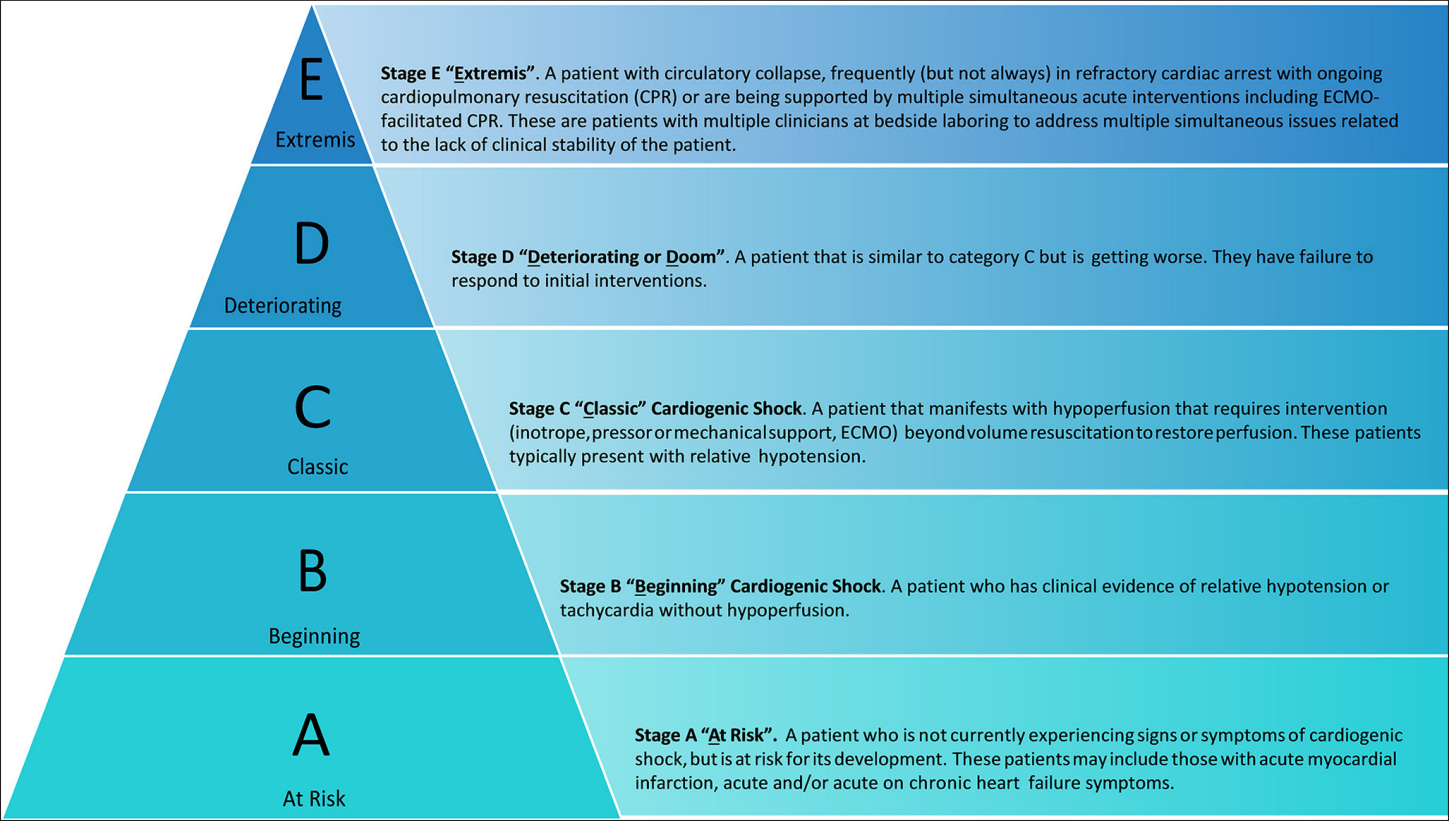
Society of Cardiovascular Angiography and Interventions (SCAI) classification of cardiogenic shock. Reprinted with permission.^28^ ECMO: extracorporeal membrane oxygenation; CPR: cardiopulmonary resuscitation

**Figure 2:**
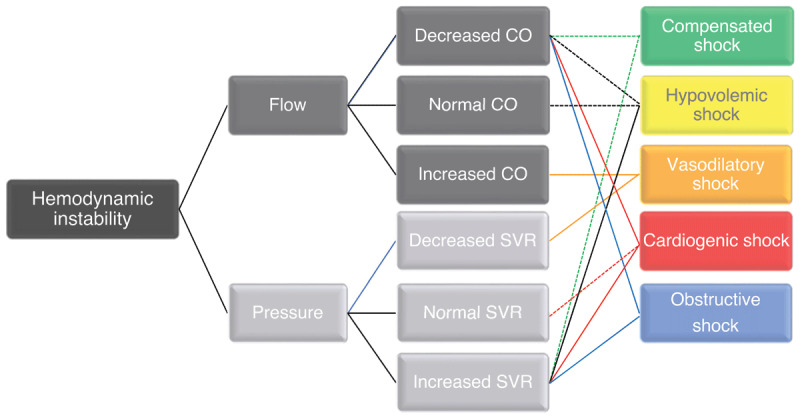
Effects of cardiac output and afterload and the inter-relationship with end-organ perfusion. CO: cardiac output; SVR: systemic vascular resistance

While initial presentation of shock includes evidence of hypoperfusion and hypotension, not all such states are equal (Figure 1).^27^ Cardiogenic shock (CS) is a continuum of hemodynamic instability, including isolated hypotension with preserved organ perfusion, isolated organ hypoperfusion despite preserved blood pressure, and the combination of hypotension and hypoperfusion.^1^ Figure 2 shows the Society for Cardiovascular Angiography & Interventions classification of CS, which aims to provide a standardized classification system across multidisciplinary teams to help provide consistent care and prognostic utility to these high-risk critically ill patients. However, further validation studies are required to determine whether the SCAI classification adds significantly to prognostication and outcomes.^28^

Hypotension and hypoperfusion are both associated with increased mortality in CICU patients. Hospital mortality is higher with isolated hypoperfusion or concomitant hypotension and hypoperfusion (classic shock); therefore, patients with hypoperfusion can be considered to have shock irrespective of blood pressure.^1^ However, the equation of MAP with markers of tissue perfusion and microcirculation per se is still up for debate.^29^ Capillary microvascular density and flow at high dose vasopressors to maintain MAP compared to normotension with no vasopressors is very different. Capillary refill time has been the subject of recent exploration and was found to be no different from lactate in guiding resuscitation in septic shock.^30^ A recent meta-analysis reported a very weak negative correlation between capillary refill time and MAP.^31^

**Figure 3:**
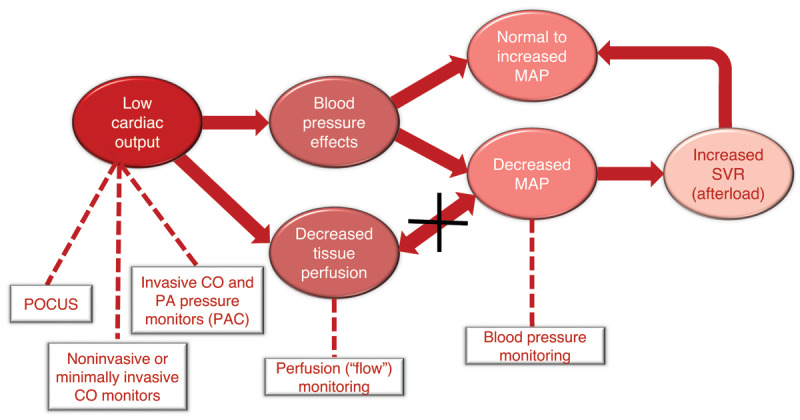
Cardiogenic shock with hypotension and hypoperfusion and compensated shock with normal or increased afterload. POCUS: point of care ultrasound; CO: cardiac output; MAP: mean arterial pressure; SVR: systemic vascular resistance; PAC: pulmonary artery catheter

The specific effects on flow and pressure can vary depending on the underlying cause and severity of the shock. In general, CS is characterized by a decrease in cardiac output, which can lead to a decrease in blood pressure. However, the relationship between flow and pressure in CS can be complex as systemic vascular resistance increases to compensate for the decreased cardiac output; this can help maintain blood pressure but may further decrease cardiac output. The goal is to optimize cardiac output and tissue perfusion by targeting a specific flow in addition to blood pressure (Figure 3). This can be assessed by measuring cardiac output directly using a pulmonary artery catheter, a minimally invasive tool that uses arterial waveform analysis with or without surface or transesophageal echocardiography (TEE) and in conjunction with trending markers of tissue hypoperfusion (Table 2).

**Table 2:** Clinical, diagnostic, and laboratory markers of tissue hypoperfusion.


1. Decreased blood pressure: systolic < 90 mm Hg OR mean pressure < 60 mm Hg OR > 30 mm Hg drop from baseline

2. Tachycardia: heart rate > 100 beats/min

3. Weak or absent peripheral pulses

4. Cool, clammy, or mottled skin

5. Altered mental status

6. Decreased urine output < 30 mL/hr

7. Cardiac index < 2.2

8. Pulmonary capillary wedge pressure > 15 mm Hg

9. Right atrial pressure ≥ 0.8 mm Hg

10. Pulmonary artery pulsatility index < 1.85

11. Cardiac power output ≤ 0.6 watts

12. Mixed venous oxygen saturation (SvO_2_) < 60%

13. Elevated lactate > 2 mmol/L

14. Creatinine doubling

15. 50% drop in glomerular filtration rate

16. Deranged liver function

17. Elevated NT-pro brain natriuretic peptide


## Monitoring and Diagnostic Modalities to Guide Blood Pressure Management in the ICU

Arterial blood pressure is determined primarily by cardiac output, systemic vascular resistance, vascular compliance, and blood volume.^32^ In the ICU, and especially in the cardiac critical care unit, use of an “invasive continuous arterial blood measurement” is widely considered the gold standard. However, blood pressure alone is simply not enough in these patients. Therefore we are seeing a consistent move away from static toward dynamic measurements.^33^ Beyond knowing that a patient is hypotensive, a critical care physician needs to determine whether the patient is fluid responsive or needs vasopressor or inotropic support. To this end, dynamic variables such as plethysmography variability index, pulse pressure variation, and stroke volume variation are helpful and increasingly used as easy bedside tools.^33^

### Plethysmography Variability Index

Plethysmography variability index (PVI) utilizes changes in pulse oximetry waveforms over the entire respiratory cycle to predict fluid responsiveness.^33^ In a prospective observational study of mechanically ventilated patients undergoing noncardiac surgery, PVI was measured and a fluid bolus given. Patients with a PVI > 10.5% were noted to have a decrease in heart rate with concurrent increase in cardiac index following the bolus.^34^ Forget et al. likewise showed that during major abdominal surgery, use of PVI to guide fluid therapies led to decreased lactate levels when compared with standard care both during surgery and 48 hours postoperatively.^35^

### Pulse Pressure Variation

Pulse pressure variation (PPV) has been a popular marker of fluid responsiveness since it requires no additional equipment beyond an invasive blood pressure monitor. To be accurate, it does require appropriately large tidal volumes in a mechanically ventilated patient using volume control ventilation in normal sinus rhythm. Yang and Du undertook a recent meta-analysis to evaluate PPV in the critical care population.^36^ This examination of 22 studies found PPV had a pooled sensitivity of 0.88 and pooled specificity of 0.89 for fluid responsiveness.^36^

### Stroke Volume Variation

Stroke volume variation (SVV) can be evaluated by one of several commercially available monitors that differ in their method of estimating aortic impedance.^37^ In a meta-analysis of 24 studies utilizing SVV in the surgical and critical care population, Zhang et al. found SVV highly correlated with fluid responsiveness (r = 0.72) and with a sensitivity and specificity of 0.81 and 0.80, respectively.^37^ The correlation was found to be even stronger in the ICU population, which the authors postulated was due to the relative hypovolemic state of these patients compared with those in the operating room when evaluated.^37^

In addition, an important and easy to use bedside tool in the ICU is the tidal volume challenge that uses the change of tidal volume from the typical lung protective 6 mL/kg to a larger target of 8 mL/kg. This change in SVV and PPV obtained by transiently increasing tidal volume (tidal volume challenge from 6 to 8 mL/kg) was superior to PPV and SVV in predicting fluid responsiveness during low tidal volume ventilation. Myatra and colleagues demonstrated this in a study in 20 patients in 2017, and these findings were validated in various larger external cohorts in subsequent analyses.^38,39^

### Echocardiography

The important role of echocardiography in the critical care setting cannot be overstated. Regardless of modality, echocardiography can be a useful tool for examining hemodynamic failure, assessing etiology of cardiopulmonary arrest, and determining volume status via superior vena cava (with transgastric TEE) or inferior vena cava (with transthoracic echocardiogram) collapsibility.^40^ In an extensive review of the literature, Prager et al. noted that utilization of TEE in the critical care setting resulted in a change of diagnosis in 52% to 78% of patients and a change in management in 32% to 79% of patients examined.^41^ They additionally showed that TEE was able to successfully distinguish the etiology of cardiac arrest in 25% to 35% of cases when it was used for this purpose.^41^

**Figure 4:**
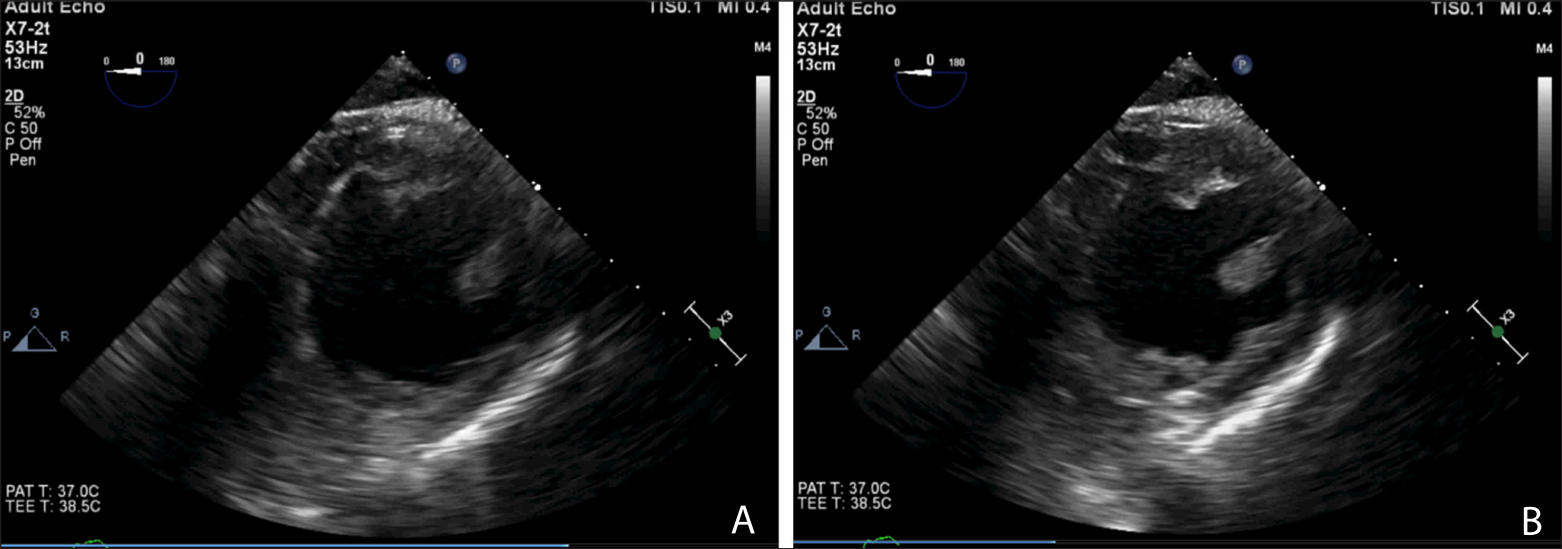
(A) Transgastric transesophageal echocardiography (TEE) short axis view of a failing left ventricle at end systole. **(B)** TEE short axis view of a failing left ventricle at end diastole. Photo credit Chandrika Garner, MD, Atrium Health Wake Forest University Medical Center

**Figure 5:**
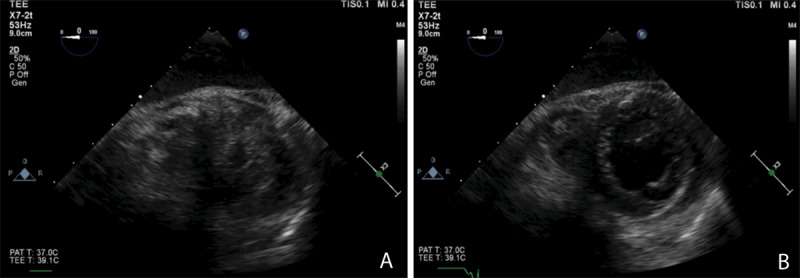
(A) Transgastric short axis transesophageal echocardiography (TEE) view of a hypertrophied, underfilled left ventricle in end systole. **(B)** Transgastric short axis TEE view of a hypertrophied, underfilled left ventricle in end diastole. Photo credit Chandrika Garner, MD, Atrium Health Wake Forest University Medical Center

**Figure 6:**
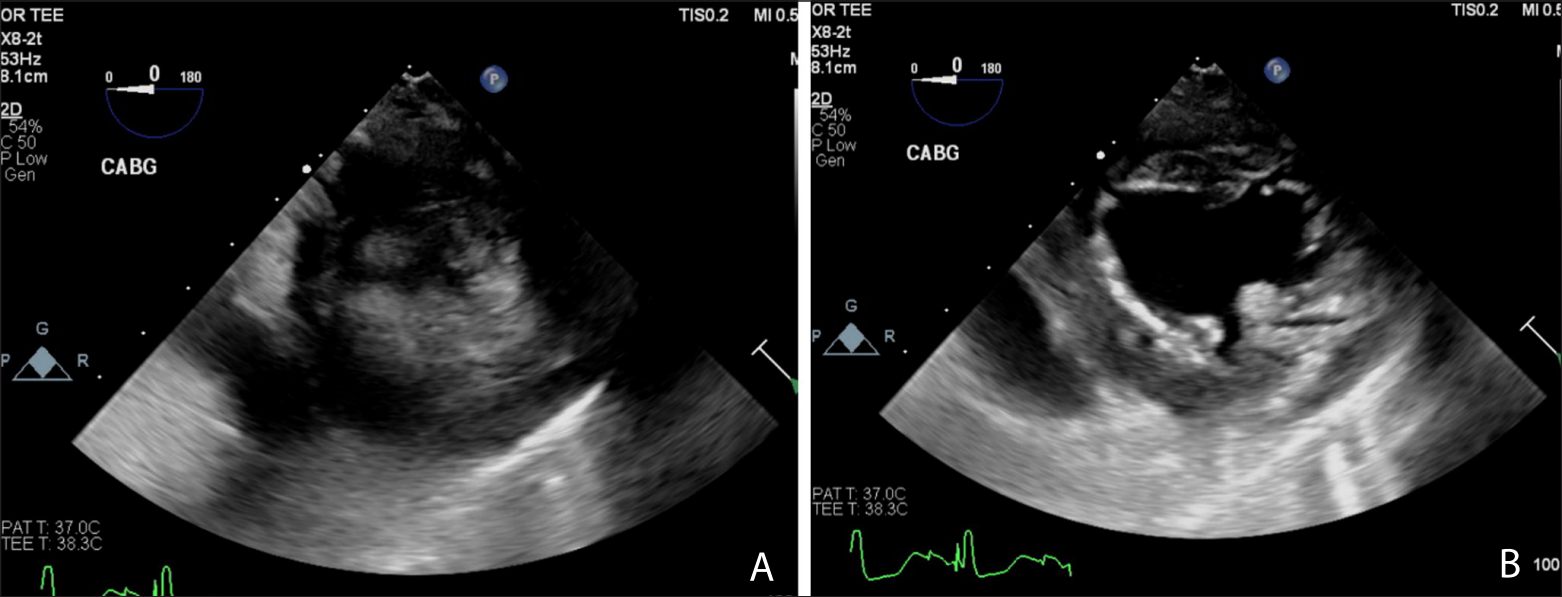
(A) Transgastric short axis transesophageal echocardiography (TEE) view of an underfilled left ventricle with normal function at end systole. This patient was hypotensive and responded to volume administration. **(B)** Transgastric short axis TEE view of an underfilled left ventricle with normal function at end diastole. Photo credit Kyle Buck, MD, Atrium Health Wake Forest Baptist Medical Center

As shown in Figures 4 A and B, minimal changes occur in the LV dimension in both systole and diastole, respectively, which is indicative of decreased LV function that can be further assessed and quantified using other 2D and 3D echocardiographic measurements. This patient responded to inotropes to increase cardiac contractility and output. Figures 5 A and B show a hypertrophied LV (septal wall thickness > 1.6 cm) with near empty LV cavity at end systole. This patient had an otherwise normal LV function and responded well hemodynamically to fluid bolus and increase in afterload. Figures 6 A and B show images of normal LV function and normal ventricular wall thickness and an underfilled LV at end systole. This patient was hypovolemic with normal cardiac function and responded to fluid bolus followed by an appropriate increase in cardiac output.

## Using Intraoperative Data to Guide Blood Pressure Management in the Critical Care Unit

Intraoperative hypotension with even brief exposure to a MAP of < 65 mm Hg is known to be associated with an increased risk of organ system failure and mortality.^7,42,43,44,45,46^ Specifically, intraoperative hypotension is associated with postoperative acute kidney injury (AKI), myocardial injury, stroke, delirium, and mortality.^7,45,47,48^ Interestingly, this relationship and risk is no different for an absolute MAP < 65 mm Hg or a relative drop from baseline by about 25%.^49^ Patients who are hypotensive in the operating room are more likely to remain hypotensive in the post-surgical ICU and possibly when they recover on the hospital ward, where it may be undetected for lack of adequate monitoring.^43,50,51^ An analysis by Khanna et al. showed that a lowest MAP reading less than that compared to a population median MAP of 87 mm Hg in the surgical ICU was significantly associated with the risk of a composite of myocardial injury or mortality. However, intraoperative hypotension had a significant interaction with the primary outcome.^52^

Even patients with normotension in the operating room may suffer harm associated with trivial new-onset hypotension in the post-surgical ICU.^8^ Unsurprisingly, in patients undergoing cardiac surgery with CPB, the total duration of intraoperative hypotension per 10-minute exposure of MAP < 65 mm Hg throughout the surgery was statistically significantly associated with the composite primary outcome of stroke, AKI, or death.^53^ Hypotension pre-CPB can result from decreased venous return, arrythmias due to surgical manipulation, depressed cardiac function, decreased systemic vascular resistance, or induced hypotension for aortic cannulation.^54^ Despite much emphasis placed on perfusion pressure, there are no guidelines for target perfusion pressure during CPB. According to the 2019 European Adult Cardiac Surgery Guidelines for CPB, it is safe to maintain MAP targets between 50 and 70 mm Hg during CPB.^55^ However, studies have shown that higher MAP targets during CPB were associated with lower post-bypass lactate levels,^56^ lower incidence of stroke,^13^ and decreased risk of AKI.^53^

It may be prudent to add monitors of cerebral perfusion and oxygenation in patients undergoing cardiac surgery, particularly in those with a history of a cerebrovascular event. A good clinical practice may be to use baseline cerebral oximetry (placed before induction of anesthesia) to monitor trends in the oximeter values with changes in blood pressure and to establish a baseline MAP goal for these patients. Subsequently, this more personalized approach could be used as a targeted postoperative MAP in the ICU to optimize cerebral perfusion pressure.

Transesophageal echocardiography is a good guide to establish volume status intraoperatively and to estimate the patient’s blood pressure goals, cardiac function, and need for pressors versus inotropes.^57^ Additionally, renal resistive index (RRI) calculated using TEE has shown that an RRI > 0.68 is a good predictor of AKI in patients undergoing cardiac surgery on CPB.^58^ An important analysis showed significant associations of intraoperative post-CPB RRI elevation with subsequent development of AKI in a cohort of 99 adult cardiac surgery patients.^59^ However, there remains a lack of evidence to support a higher MAP target in patients with increased baseline RRI, who are presumably at high risk for postoperative AKI and mortality.

## Looking to the Future

The latest iteration of blood pressure evaluation pivots from dynamic measurements to prediction and proactive management of hypotension. This is a step beyond our traditional management of hypotension based on reactive responses, which result in time delays of various lengths and may not be adequate to prevent downstream effects.

Machine-learning-derived algorithms that predict hypotension are based on arterial wave form analysis using millions of waveforms from various shock pathophysiological states in model development and external validation.^60,61^ One such algorithm can predict arterial hypotension with high sensitivity and specificity.^61^ Davies et al. analyzed this algorithm in 255 patients undergoing major noncardiac surgery and determined a sensitivity and specificity of about 80% 15 minutes preceding hypotension and 85% five minutes prior.^62^ A recent systematic review by Li et al. evaluated a total of five trials of the hypotension predictive index and determined that it has the potential to reduce frequency and severity of hypotensive episodes when strictly adhering to the protocol.^60^

Most data surrounding these technologies come from the operating room and support the thought that different varieties of intraoperative hypotension need different treatment, an idea which also could be extrapolated to the ICU within limits.^63,64^ Randomized trials have reported conflicting evidence for benefits, which may be due to variable adherence to protocol and directions from technology.^65,66^ Ongoing trials, including the HPI (Hypotension Prediction Index) CARE Trial, are currently underway to examine this technology in cardiac surgery and cardiac critical care.^67^ Other trials, such as the REACT-SHOCK group in Australia and New Zealand, will examine targeted BP management in the ICU based on baseline (pre-morbid) pressures. Previously, important data suggested that we tend to keep patients with lower pre-morbid pressures on a longer duration of vasopressors, with consequently longer ICU stays, all of which may be unnecessary.^68^ This and the use of perfusion pressure targets for the future of large interventional pressure targeted trials will be necessary as we grow the evidence base.

## Conclusion

We have yet to establish a definitive target for hypotension in different clinical settings. This should prompt us to move away from using a single predefined number for a hypotensive threshold for all patients and towards using a more individualized approach based on patient characteristics. Further, we should consider integrating emerging innovative technologies to assess and monitor an individual patient’s hemodynamic profile, using parameters in addition to blood pressure, before and during admission to a critical care unit. The goal is to optimize tissue perfusion and oxygen delivery rather than targeting a single blood pressure goal as we advance and personalize care for the critically ill.

## Key Points

- Individualization of mean arterial pressure (MAP) goals is important in the post-cardiac surgery patient, as different comorbidities, operative interventions, and the presence of mechanical circulatory support, amongst other criteria, may influence the MAP required to maintain adequate perfusion.
- Almost 10 years after the impressive work by the SEPSISPAM (Sepsis and Mean Arterial Pressure) group, we need to think of trials with appropriate patient populations, targets, and outcomes, as studies have shown that certain vulnerable patient populations may need a higher blood pressure target.
- Other blood pressure components, namely mean, systolic, and diastolic pressures, are comparable in their strength of association with organ system injury and intensive care unit (ICU) mortality.
- Hospital outcomes, including mortality, are worse in patients with evidence of both hypotension and hypoperfusion.
- Analysis comparing perfusion pressure deficits and MAP deficits have been seen to have a nearly similar and strong association with ICU mortality. This is a critical area of focus.
- As intensivists, we should move away from a single predefined blood pressure target for all patients and toward a more individualized approach based on patient characteristics.

## CME Credit Opportunity

Houston Methodist is accredited by the Accreditation Council for Continuing Medical Education (ACCME) to provide continuing medical education for physicians.

Houston Methodist designates this Journal-based CME activity for a maximum of *1 AMA PRA Category 1 Credit*™. Physicians should claim only the credit commensurate with the extent of their participation in the activity.

Click to earn CME credit: learn.houstonmethodist.org/MDCVJ-19.4.

## References

[1] Jentzer JC, Burstein B, Van Diepen S, et al. Defining Shock and Preshock for Mortality Risk Stratification in Cardiac Intensive Care Unit Patients. Circ Heart Fail. 2021 Jan;14(1):e007678. doi: 10.1161/CIRCHEARTFAILURE.120.007678 33464952

[2] Maheshwari K, Nathanson BH, Munson SH, et al. The relationship between ICU hypotension and in-hospital mortality and morbidity in septic patients. Intensive Care Med. 2018 Jun;44(6):857-867. doi: 10.1007/s00134-018-5218-5 29872882 PMC6013508

[3] Evans L, Rhodes A, Alhazzani W, et al. Surviving Sepsis Campaign: International Guidelines for Management of Sepsis and Septic Shock 2021. Crit Care Med. 2021 Nov 1;49(11):e1063-e1143. doi: 10.1097/CCM.0000000000005337 34605781

[4] Asfar P, Meziani F, Hamel JF, et al. High versus low blood-pressure target in patients with septic shock. N Engl J Med. 2014 Apr 24;370(17):1583-93. doi: 10.1056/NEJMoa1312173 24635770

[5] Khanna AK, Kinoshita T, Natarajan A, et al. Association of systolic, diastolic, mean, and pulse pressure with morbidity and mortality in septic ICU patients: a nationwide observational study. Ann Intensive Care. 2023 Feb 20;13(1):9. doi: 10.1186/s13613-023-01101-4 36807233 PMC9941378

[6] Khanna AK, Maheshwari K, Mao G, et al. Association Between Mean Arterial Pressure and Acute Kidney Injury and a Composite of Myocardial Injury and Mortality in Postoperative Critically Ill Patients: A Retrospective Cohort Analysis. Crit Care Med. 2019 Jul;47(7):910-917. doi: 10.1097/CCM.0000000000003763 30985388

[7] Maheshwari K, Ahuja S, Khanna AK, et al. Association Between Perioperative Hypotension and Delirium in Postoperative Critically Ill Patients: A Retrospective Cohort Analysis. Anesth Analg. 2020 Mar;130(3):636-643. doi: 10.1213/ANE.0000000000004517 31725024

[8] Smischney NJ, Shaw AD, Stapelfeldt WH, et al. Postoperative hypotension in patients discharged to the intensive care unit after non-cardiac surgery is associated with adverse clinical outcomes. Crit Care. 2020 Dec 7;24(1):682. doi: 10.1186/s13054-020-03412-5 33287872 PMC7720547

[9] Jouan Y, Seegers V, Meziani F, et al. Effects of mean arterial pressure on arousal in sedated ventilated patients with septic shock: a SEPSISPAM post hoc exploratory study. Ann Intensive Care. 2019 May 9;9(1):54. doi: 10.1186/s13613-019-0528-5 31073873 PMC6509319

[10] Lamontagne F, Richards-Belle A, Thomas K, et al. Effect of Reduced Exposure to Vasopressors on 90-Day Mortality in Older Critically Ill Patients With Vasodilatory Hypotension: A Randomized Clinical Trial. JAMA. 2020 Mar 10;323(10):938-949. doi: 10.1001/jama.2020.0930 32049269 PMC7064880

[11] McEvoy JW, Chen Y, Rawlings A, et al. Diastolic Blood Pressure, Subclinical Myocardial Damage, and Cardiac Events: Implications for Blood Pressure Control. J Am Coll Cardiol. 2016 Oct 18;68(16):1713-1722. doi: 10.1016/j.jacc.2016.07.754 27590090 PMC5089057

[12] Burstein B, Tabi M, Barsness GW, Bell MR, Kashani K, Jentzer JC. Association between mean arterial pressure during the first 24 hours and hospital mortality in patients with cardiogenic shock. Crit Care. 2020 Aug 20;24(1):513. doi: 10.1186/s13054-020-03217-6 32819421 PMC7439249

[13] Sun LY, Chung AM, Farkouh ME, et al. Intraoperative Hypotension and Acute Kidney Injury, Stroke, and Mortality during and outside Cardiopulmonary Bypass: A Retrospective Observational Cohort Study. Anesthesiology. 2022 Jun 1;136(6):927-939. doi: 10.1097/ALN.0000000000004175 35188970

[14] de la Hoz MA, Rangasamy V, Bastos AB, et al. Intraoperative Hypotension and Acute Kidney Injury, Stroke, and Mortality during and outside Cardiopulmonary Bypass: A Retrospective Observational Cohort Study. Anesthesiology. 2022 Jun 1;136(6):927-939. doi: 10.1097/ALN.0000000000004175 35188970

[15] Sato R, Duggal A, Sacha GL, et al. The Relationship Between Norepinephrine Equivalent Dose of Vasopressors Within 24 Hours From the Onset of Septic Shock and In-Hospital Mortality Rate. Chest. 2023 Jan;163(1):148-151. doi: 10.1016/j.chest.2022.07.018 35921884

[16] Wieruszewski PM, Khanna AK. Vasopressor Choice and Timing in Vasodilatory Shock. Crit Care. 2022 Mar 22;26(1):76. doi: 10.1186/s13054-022-03911-7 35337346 PMC8957156

[17] Asfar P, Radermacher P, Ostermann M. MAP of 65: target of the past? Intensive Care Med. 2018 Sep;44(9):1551-1552. doi: 10.1007/s00134-018-5292-8 30003302

[18] Panwar R. Untreated Relative Hypotension Measured as Perfusion Pressure Deficit During Management of Shock and New-Onset Acute Kidney Injury-A Literature Review. Shock. 2018 May;49(5):497-507. doi: 10.1097/SHK.0000000000001033 29040214

[19] Panwar R, Tarvade S, Lanyon N, et al. Relative Hypotension and Adverse Kidney-related Outcomes among Critically Ill Patients with Shock. A Multicenter, Prospective Cohort Study. Am J Respir Crit Care Med. 2020 Nov 15;202(10):1407-1418. doi: 10.1164/rccm.201912-2316OC 32614244

[20] Meng L, Yu W, Wang T, Zhang L, Heerdt PM, Gelb AW. Blood Pressure Targets in Perioperative Care. Hypertension. 2018 Oct;72(4):806-817. doi: 10.1161/HYPERTENSIONAHA.118.11688 30354725

[21] Mathew R, Fernando SM, Hu K, et al. Optimal Perfusion Targets in Cardiogenic Shock. JACC: Advances. 2022 Jun;1(2):100034. doi: 10.1016/j.jacadv.2022.100034 PMC11198174 38939320

[22] Ameloot K, Jakkula P, Hästbacka J, et al. Optimum Blood Pressure in Patients With Shock After Acute Myocardial Infarction and Cardiac Arrest. J Am Coll Cardiol. 2020 Aug 18;76(7):812-824. doi: 10.1016/j.jacc.2020.06.043 32792079

[23] Bootsma IT, de Lange F, Scheeren TWL, Jainandunsing JS, Boerma EC. High Versus Normal Blood Pressure Targets in Relation to Right Ventricular Dysfunction After Cardiac Surgery: A Randomized Controlled Trial. J Cardiothorac Vasc Anesth. 2021 Oct;35(10):2980-2990. doi: 10.1053/j.jvca.2021.02.054 33814247

[24] van Blankenstein JH, Slager CJ, Soei LK, Boersma H, Verdouw PD. Effect of arterial blood pressure and ventilation gases on cardiac depression induced by coronary air embolism. J Appl Physiol (1985). 1994 Oct;77(4):1896-902. doi: 10.1152/jappl.1994.77.4.1896 7836215

[25] Hori D, Katz NM, Fine DM, et al. Defining oliguria during cardiopulmonary bypass and its relationship with cardiac surgery-associated acute kidney injury. Br J Anaesth. 2016 Dec;117(6):733-740. doi: 10.1093/bja/aew340 27956671 PMC5155559

[26] McIlroy D, Murphy D, Kasza J, Bhatia D, Marasco S. Association of postoperative blood pressure and bleeding after cardiac surgery. J Thorac Cardiovasc Surg. 2019 Nov;158(5):1370-1379.e6. doi: 10.1016/j.jtcvs.2019.01.063 30853233

[27] Khorsand S, Helou MF, Satyapriya V, Kopanczyk R, Khanna AK. Not all Shock States Are Created Equal: A Review of the Diagnosis and Management of Septic, Hypovolemic, Cardiogenic, Obstructive, and Distributive Shock. Anesthesiol Clin. 2023 Mar;41(1):1-25. doi: 10.1016/j.anclin.2022.11.002 36871993

[28] Baran DA, Grines CL, Bailey S, et al. SCAI clinical expert consensus statement on the classification of cardiogenic shock: This document was endorsed by the American College of Cardiology (ACC), the American Heart Association (AHA), the Society of Critical Care Medicine (SCCM), and the Society of Thoracic Surgeons (STS) in April 2019. Catheter Cardiovasc Interv. 2019 Jul 1;94(1):29-37. doi: 10.1002/ccd.28329 31104355

[29] Khanna AK, Karamchandani K. Macrocirculation and Microcirculation: The “Batman and Superman” Story of Critical Care Resuscitation. Anesth Analg. 2021 Jan;132(1):280-283. doi: 10.1213/ANE.0000000000005272 33177325

[30] Hernández G, Ospina-Tascón GA, Damiani LP, et al. Effect of a Resuscitation Strategy Targeting Peripheral Perfusion Status vs Serum Lactate Levels on 28-Day Mortality Among Patients With Septic Shock: The ANDROMEDA-SHOCK Randomized Clinical Trial. JAMA. 2019 Feb 19;321(7):654-664. doi: 10.1001/jama.2019.0071 30772908 PMC6439620

[31] Putowski Z, Goldyn M, Pluta MP, Krzych LJ, Hernandez G, Kattan E. Correlation Between Mean Arterial Pressure and Capillary Refill Time in Patients with Septic Shock: A Systematic Review and Meta-analysis. J Intensive Care Med. 2023 Apr 11;8850666231168038. doi: 10.1177/08850666231168038 37042043

[32] Briesenick L, Flick M, Saugel B. Postoperative blood pressure management in patients treated in the ICU after noncardiac surgery. Curr Opin Crit Care. 2021 Dec 1;27(6):694-700. doi: 10.1097/MCC.0000000000000884 34757996

[33] Scheeren TWL, Ramsay MAE. New Developments in Hemodynamic Monitoring. J Cardiothorac Vasc Anesth. 2019 Aug;33 Suppl 1:S67-S72. doi: 10.1053/j.jvca.2019.03.043 31279355

[34] Siswojo AS, Wong DM-Y, Phan TD, Kluger R. Pleth variability index predicts fluid responsiveness in mechanically ventilated adults during general anesthesia for noncardiac surgery. J Cardiothorac Vasc Anesth. 2014 Dec;28(6):1505-9. doi: 10.1053/j.jvca.2014.04.010 25169895

[35] Forget P, Lois F, de Kock M. Goal-directed fluid management based on the pulse oximeter-derived pleth variability index reduces lactate levels and improves fluid management. Anesth Analg. 2010 Oct;111(4):910-4. doi: 10.1213/ANE.0b013e3181eb624f 20705785

[36] Yang X, Du B. Does pulse pressure variation predict fluid responsiveness in critically ill patients? A systematic review and meta-analysis. Crit Care. 2014 Nov 27;18(6):650. doi: 10.1186/s13054-014-0650-6 25427970 PMC4258282

[37] Zhang Z, Lu B, Sheng X, Jin N. Accuracy of stroke volume variation in predicting fluid responsiveness: a systematic review and meta-analysis. J Anesth. 2011 Dec;25(6):904-16. doi: 10.1007/s00540-011-1217-1 21892779

[38] Myatra SN, Prabu NR, Divatia JV, Monnet X, Kulkarni AP, Teboul JL. The Changes in Pulse Pressure Variation or Stroke Volume Variation After a «Tidal Volume Challenge» Reliably Predict Fluid Responsiveness During Low Tidal Volume Ventilation. Crit Care Med. 2017 Mar;45(3):415-421. doi: 10.1097/CCM.0000000000002183 27922879

[39] Myatra SN, Monnet X, Teboul JL. Use of ‘tidal volume challenge’ to improve the reliability of pulse pressure variation. Crit Care. 2017 Mar 21;21(1):60. doi: 10.1186/s13054-017-1637-x 28320434 PMC5359814

[40] Mayo PH, Narasimhan M, Koenig S. Critical Care Transesophageal Echocardiography. Chest. 2015 Nov;148(5):1323-1332. doi: 10.1378/chest.15-0260 26204465

[41] Prager R, Bowdridge J, Pratte M, Cheng J, McInnes MD, Arntfield R. Indications, Clinical Impact, and Complications of Critical Care Transesophageal Echocardiography: A Scoping Review. J Intensive Care Med. 2023 Mar;38(3):245-272. doi: 10.1177/08850666221115348 35854414 PMC9806486

[42] Gregory A, Stapelfeldt WH, Khanna AK, et al. Intraoperative Hypotension Is Associated With Adverse Clinical Outcomes After Noncardiac Surgery. Anesth Analg. 2021 Jun 1;132(6):1654-1665. doi: 10.1213/ANE.0000000000005250 33177322 PMC8115733

[43] Khanna AK, Shaw AD, Stapelfeldt WH, et al. Postoperative Hypotension and Adverse Clinical Outcomes in Patients Without Intraoperative Hypotension, After Noncardiac Surgery. Anesth Analg. 2021 May 1;132(5):1410-1420. doi: 10.1213/ANE.0000000000005374 33626028

[44] Shaw AD, Khanna AK, Smischney NJ, et al. Intraoperative hypotension is associated with persistent acute kidney disease after noncardiac surgery: a multicentre cohort study. Br J Anaesth. 2022 Jul;129(1):13-21. doi: 10.1016/j.bja.2022.03.027 35595549

[45] Sessler DI, Khanna AK. Perioperative myocardial injury and the contribution of hypotension. Intensive Care Med. 2018 Jun;44(6):811-822. doi: 10.1007/s00134-018-52?24-7 29868971

[46] Stapelfeldt WH, Khanna AK, Shaw AD, et al. Association of perioperative hypotension with subsequent greater healthcare resource utilization. J Clin Anesth. 2021 Dec;75:110516. doi: 10.1016/j.jclinane.2021.110516 34536719

[47] Walsh M, Devereaux PJ, Garg AX, et al. Relationship between intraoperative mean arterial pressure and clinical outcomes after noncardiac surgery: toward an empirical definition of hypotension. Anesthesiology. 2013 Sep;119(3):507-15. doi: 10.1097/ALN.0b013e3182a?10e26 23835589

[48] Maheshwari K, Turan A, Mao G, et al. The association of hypotension during non-cardiac surgery, before and after skin incision, with postoperative acute kidney injury: a retrospective cohort analysis. Anaesthesia. 2018 Oct;73(10):1223-1228. doi: 10.1111/anae.14416 30144029

[49] Salmasi V, Maheshwari K, Yang D, et al. Relationship between Intraoperative Hypotension, Defined by Either Reduction from Baseline or Absolute Thresholds, and Acute Kidney and Myocardial Injury after Noncardiac Surgery: A Retrospective Cohort Analysis. Anesthesiology. 2017 Jan;126(1):47-65. doi: 10.1097/ALN.0000000000001432 27792044

[50] Shimada T, Cohen B, Shah K, et al. Associations between intraoperative and post-anesthesia care unit hypotension and surgical ward hypotension. J Clin Anesth. 2021 Dec;75:110495. doi: 10.1016/j.jclinane.2021.110495 34560444

[51] Turan A, Chang C, Cohen B, et al. Incidence, Severity, and Detection of Blood Pressure Perturbations after Abdominal Surgery: A Prospective Blinded Observational Study. Anesthesiology. 2019 Apr;130(4):550-559. doi: 10.1097/ALN.0000000000002626 30875354

[52] Khanna AK, Maheshwari K, Mao G, et al. Association Between Mean Arterial Pressure and Acute Kidney Injury and a Composite of Myocardial Injury and Mortality in Postoperative Critically Ill Patients: A Retrospective Cohort Analysis. Crit Care Med. 2019 Jul;47(7):910-917. doi: 10.1097/CCM.0000000000003763 30985388

[53] de la Hoz MA, Rangasamy V, Bastos AB, et al. Intraoperative Hypotension and Acute Kidney Injury, Stroke, and Mortality during and outside Cardiopulmonary Bypass: A Retrospective Observational Cohort Study. Anesthesiology. 2022 Jun 1;136(6):927-939. doi: 10.1097/ALN.0000000000004175 35188970

[54] Hogue CW, Gottesman RF, Stearns J. Mechanisms of cerebral injury from cardiac surgery. Crit Care Clin. 2008 Jan;24(1):83-98, viii-ix. doi: 10.1016/j.ccc.2007.09.004 18241780 PMC2276597

[55] Kunst G, Milojevic M, Boer C, et al. 2019 EACTS/EACTA/EBCP guidelines on cardiopulmonary bypass in adult cardiac surgery. Br J Anaesth. 2019 Dec;123(6):713-757. doi: 10.1016/j.bja.2019.09.012 31585674

[56] Miao Q, Wu DJ, Chen X, et al. Target blood pressure management during cardiopulmonary bypass improves lactate levels after cardiac surgery: a randomized controlled trial. BMC Anesthesiol. 2021 Dec 8;21(1):309. doi: 10.1186/s12871-021-01537-w PMC8653567 34879822

[57] Schulmeyer C, Farías J, Rajdl E, de La Maza J, Labbé M. Utility of transesophageal echocardiography during severe hypotension in non-cardiac surgery. Rev Bras Anestesiol. 2010 Sep-Oct;60(5):513-21. doi: 10.1016/S0034-7094(10)70062-3 20863931

[58] Kajal K, Chauhan R, Negi SL, et al. Intraoperative evaluation of renal resistive index with transesophageal echocardiography for the assessment of acute renal injury in patients undergoing coronary artery bypass grafting surgery: A prospective observational study. Ann Card Anaesth. 2022 Apr-Jun;25(2):158-163. doi: 10.4103/aca.aca_221_20 35417961 PMC9244272

[59] Cherry AD, Hauck JN, Andrew BY, et al. Intraoperative renal resistive index threshold as an acute kidney injury biomarker. J Chin Anesth. 2020 May;61:109626. doi: 10.1016/j.jclinane.2019.109626 PMC6962557 31699495

[60] Li W, Hu Z, Yuan Y, Liu J, Li K. Effect of hypotension prediction index in the prevention of intraoperative hypotension during noncardiac surgery: A systematic review. J Clin Anesth. 2022 Dec;83:110981. doi: 10.1016/j.jclinane.2022.110981 36242978

[61] Hatib F, Jian Z, Buddi S, et al. Machine-learning Algorithm to Predict Hypotension Based on High-fidelity Arterial Pressure Waveform Analysis. Anesthesiology. 2018 Oct;129(4):663-674. doi: 10.1097/ALN.0000000000002300 29894315

[62] Davies SJ, Vistisen ST, Jian Z, Hatib F, Scheeren TWL. Ability of an Arterial Waveform Analysis-Derived Hypotension Prediction Index to Predict Future Hypotensive Events in Surgical Patients. Anesth Analg. 2020 Feb;130(2):352-359. doi: 10.1213/ANE.0000000000004121 30896602

[63] Khanna AK, Zarbock A, Legrand M. All intraoperative hypotension is not created equal – A call for an individualized approach. J Clin Anesth. 2023 Aug;87:111076. doi: 10.1016/j.jclinane.2023.111076 36889147

[64] Legrand M, Zarbock A. Ten tips to optimize vasopressors use in the critically ill patient with hypotension. Intensive Care Med. 2022 Jun;48(6):736-739. doi: 10.1007/s00134-022-06708-y 35504977

[65] Wijnberge M, Geerts BF, Hol L, et al. Effect of a Machine Learning-Derived Early Warning System for Intraoperative Hypotension vs Standard Care on Depth and Duration of Intraoperative Hypotension During Elective Noncardiac Surgery: The HYPE Randomized Clinical Trial. JAMA. 2020 Mar 17;323(11):1052-1060. doi: 10.1001/jama.2020.0592 32065827 PMC7078808

[66] Maheshwari K, Shimada T, Yang D, et al. Hypotension Prediction Index for Prevention of Hypotension during Moderate- to High-risk Noncardiac Surgery. Anesthesiology. 2020 Dec 1;133(6):1214-1222. doi: 10.1097/ALN.0000000000003557 32960954

[67] Rellum SR, Schuurmans J, van der Ven WH, et al. Machine learning methods for perioperative anesthetic management in cardiac surgery patients: a scoping review. J Thorac Dis. 2021 Dec;13(12):6976-6993. doi: 10.21037/jtd-21-765 35070381 PMC8743411

[68] Gershengorn HB, Stelfox HT, Niven DJ, Wunsch H. Association of Premorbid Blood Pressure with Vasopressor Infusion Duration in Patients with Shock. Am J Respir Crit Care Med. 2020 Jul 1;202(1):91-99. doi: 10.1164/rccm.201908-1681OC 32272020

